# Breed, sex, and litter effects in 2-month old puppies’ behaviour in a standardised open-field test

**DOI:** 10.1038/s41598-017-01992-x

**Published:** 2017-05-11

**Authors:** Shanis Barnard, Sarah Marshall-Pescini, Annalisa Pelosi, Chiara Passalacqua, Emanuela Prato-Previde, Paola Valsecchi

**Affiliations:** 1 Dipartimento di Scienze Chimiche, della Vita e della Sostenibilità Ambientale, Università degli Studi di Parma, Parma, Italy; 2 School of Psychology, Queen’s University Belfast, Belfast, UK; 3 Comparative Cognition, Messerli Research Institute, University of Veterinary Medicine, Vienna, Medical University of Vienna, University of Vienna, Vienna, Austria; 4 Wolf Science Centre, Ernstbrunn, Austria; 5 Dipartimento di Medicina e Chirurgia, Università degli Studi di Parma, Parma, Italy; 6 Dipartimento di Fisiopatologia Medico-Chirurgica e dei Trapianti, Sezione di Neuroscienze, Università di Milano, Milano, Italy

## Abstract

A considerable number of studies have reported differences among dog breeds with respect to their genetic profile, cognitive abilities or personality traits. Each dog breed is normally treated as a homogeneous group, however, researchers have recently questioned whether the behavioural profile of modern breeds still reflects their historical function or if the intense divergent selective pressures and geographical barriers have created a more fragmented picture. The majority of studies attempting to assess and compare modern breeds’ personality focused on the evaluation of adult dogs where the potential effects of environmental/human factors on the dogs’ behaviour are hard to discern from their genetic heritage. In the following study, we aimed at investigating between- and within-breed differences in the personality of two-months-old puppies by direct behavioural observation of 377 puppies from 12 breeds. Results showed that there was no effect of sex, however both breed and litter, significantly affected all personality traits. Breed on average explained 10% of the variance, whereas the effect of litter was noticeably higher, explaining on average 23% of the variance. Taken together, our results suggest that breed does have some influence on personality traits, but they also highlight the importance of taking litter effects into account.

## Introduction

The assessment of dogs’ personality has gained increasing attention in the last decades for its potential applicability^1–4^, as well as for more theoretical aspects, such as the genetic basis of complex behaviour^5, 6^. One of the topics, strictly linked to dog personality that is still being debated, is whether dogs from different breeds significantly diverge on specific behavioural traits, potentially reflecting their historical function (original selection to optimise their performance in specific tasks as stated by the kennel clubs standards)^7–9^. Breed profiling has largely been based on historical and anecdotal notions rather than scientific evidence^8^. However, more recently scientific attempts to provide reliable profiling of modern breeds, taking into account the large within-breed variability due to different selective pressures and geographical barriers, have been carried out^7, 8, 10–12^. Svartberg^7^, for example, found that within the same breeds, irrespective of their historical function, individuals in lines selected for companionship showed high levels of playfulness, whereas selection for use in dog shows correlated positively with fearfulness and negatively with playfulness, aggressiveness, and curiosity. However, thus far, the majority of studies attempting to assess breed personality have been carried out using questionnaire-based methods^8, 13, 14^ and have largely focused on the evaluation of adult dogs^7, 9^. Yet, behavioural variability towards different stimuli and situations can be detected also in puppies^15–18^ and arguably, if there are breed differences in temperament, observing these in puppies would provide stronger evidence, since the potential effects of environmental and human factors on the dogs’ behaviour would be minimized when compared to adult animals.

In the following study we aimed at investigating the role of sex, litter and breed on the personality of puppies at two months of age. Indeed, to our knowledge, there are no studies assessing the relative weight of these factors on the expression of personality traits in in young pups. Consequently, we assessed personality by direct behavioural observations of 377 two-months-old puppies from 12 different breeds using a standardised and previously validated open-field test^15^, in which pups were simultaneously exposed to a number of different novel stimuli (e.g. a mirror, a squeaky toy, a child-sized doll) and an unfamiliar person. We investigated whether breed differences would emerge in the pups’ behaviour, once potential sex and litter effects were accounted for.

## Results

### Cluster Analysis

**Figure 1:**
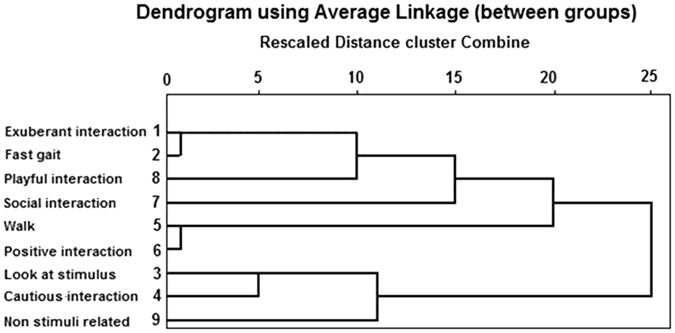
Agglomeration dendrogram. The branching-type graph illustrates the results of the Hierarchical Cluster Analysis. To find which variables are clustering at a given step, trace backwards down the branches to the variable name. The 0 to 25 scale along the top of the chart is a measure of the relative distance between clusters. The bigger the distances before two clusters are joined, the bigger the differences between these clusters.

The analysis extracted six clusters (Fig. 1 and Table S1) comparable to those found previously in Barnard, Marshall-Pescini *et al*.^15^ At the first agglomeration stadium the analysis shows three clusters: CL1. exuberant approach/interaction and fast gait labelled “Exuberant attitude”, CL2. looking at stimuli and cautious approach/interaction labelled “Cautious attitude” and CL3. walking and positive approach/interaction labelled “Relaxed attitude”. The other three variables remained as single items until a later stadium and as the measure of the relative distance was very high (see agglomeration coefficients in Table S1) these were treated as individual clusters (i.e. CL4. Social interaction, CL5. Playful interaction and CL6. Non-stimuli related behaviour).

### Breed, Litter and Sex effect on personality traits

Following a dredge selection procedure on nested data (see details in the analyses section below), adding the breed as fixed factor improved the null model fit for all personality clusters (Table 1), whereas sex did not show significant effects, neither as a single factor nor in additive and interaction models with breed (Table S3). Moreover, the litter factor resulted in better models than both the null and breed models. All models merging litter, breed and sex (litter/breed, sex*litter, sex + litter) were flawed by rank deficiency and thus showed an inadequate fit to data (see Tables S2 and S3).

**Table 1:** GLMM to determine the effect of litter and breed on each cluster group.

Cluster	Litter	df	F	p-value	Marginal R Sq.
CL1_Exuberant attitude	litter	73	5.42	<0.001	0.238
	breed	11	2.92	<0.001	0.083
CL2_Cautious attitude	litter	73	3.23	<0.001	0.241
	breed	11	5.14	<0.001	0.125
CL3_Relaxed attitude	litter	73	3.25	<0.001	0.242
	breed	11	3.08	<0.001	0.105
CL4_Social interaction	litter	73	2.22	<0.001	0.179
	breed	11	2.86	0.001	0.114
CL5_Playful	litter	73	2.67	<0.001	0.207
	breed	11	2.87	0.001	0.094
CL6_Non stimuli related	litter	73	3.84	<0.001	0.274
	breed	11	1.89	0.04	0.056

Only significant factors are shown here, marginal R squared represents the explained variance of each fixed factor.

Breed explained between 6–12% of the total variance whereas the litter explained between 18–27% of the variance. Considering the values of the marginal R squared, are double for litter compared to breed in all traits except for the social interaction with humans (Table 1); the effect of litter emerges as playing a stronger role than breed in modulating puppies’ behaviours.

**Figure 2:**
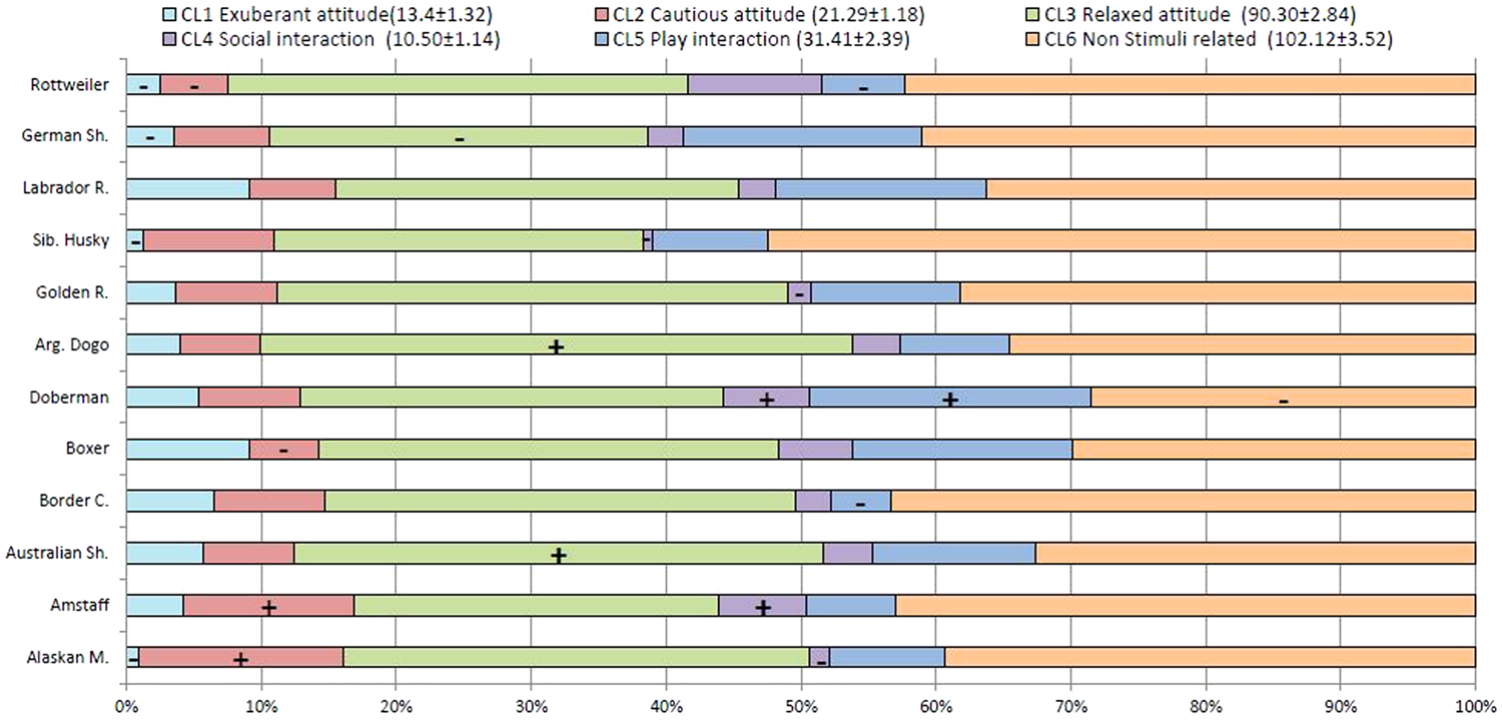
Breed behavioural profiles. Bars show the proportion of expression of the six personality traits for each breed. *Legend*: in brackets the population mean ± SEM for each personality traits. Minus and plus inside bars indicate values that are significantly below or above the population mean respectively (α ≤ 0.05). For each trait, mean values and statistics are available in Table S4.

To explore between breed differences, each breed was compared to the population mean for any given trait (Fig. 2, full statistical reporting in Table S4). For example, we found that: American staffordshire puppies were significantly less playful and more cautious than the average population; Siberian husky and Alaskan malamute spent significantly less time than average in exuberant and social interactions and Alaskan puppies were also more cautious than average. Furthermore, Border collies were significantly less playful and Boxers significantly less cautious than average. Finally, Doberman puppies spent less time than average focusing on the environment but more time playing in the arena and socially engaging with people.

## Discussion

In the current study, we assessed the personality of 2-months-old puppies pertaining to 12 different breeds by direct observation of their behaviour in a modified open-field test. Confirming previous results^15^ and thereby adding robustness to our assessment tool, the cluster analysis extracted six main personality traits “Exuberant attitude”, “Cautious attitude”, “Relaxed attitude”, “Social interaction”, “Playful interaction” and “Non-stimuli related behaviour”.

By adopting a model selection procedure, we assessed the effect of sex, breed and litter on the expression of these traits. We found no significant effect of sex, a moderate effect of breed and a strong effect of litter, the latter explaining the highest proportion of variability for all personality traits. To our knowledge, this is the first study that simultaneously takes into account both breed and litter effects when analysing puppy personality. The large variability both within and across breeds found here, mirrors results with adult dogs. For example, Björnerfeldt *et al*.^10^ found that in poodles, intra-breed genetic differentiation can be as strong as between-breed differentiation. Nevertheless, despite the strong and expected litter effect, breed also played a significant role in the expression of personality traits.

It is well documented that dog breeds differ from one another genetically^19–21^, behaviourally^9, 13^ and cognitively^16^ and, more specifically, a number of studies have also shown significant breed difference in the temperament/personality of adult dogs^8, 22^. Our results extend this research by showing that breed differences can be detected as early as 2-months of age and are in line with Scott & Fuller’s^23^ pioneering studies, where marked behavioural differences were shown to occur during early development in puppies of different breeds raised under identical conditions. For example, during an arena test at 4 months-old, wirehaired terrier puppies were significantly more active and aggressive than the calmer beagles^24^. It is worth noting, however, that in our study breed counts for less than 10% of the explained variance whereas the effect of litter explained on average 23% of the total variance (Table 1).

In recent decades there has been an outburst of divergent selective forces within breeds, e.g. selection for morphology (i.e. size), behaviour, working or show purposes, as well as the effect of geographic isolation^10, 12^. This fragmentation has led to genetically differentiated types often ignored in comparative breed studies, where a breed is normally considered as a relatively homogeneous group^10^. Thus, a strain dependent genetic effect may partially explain these pronounced within-breed differences.

Furthermore, previous research on a population of German Shepherds, reported that factors such as litter size, sex ratio, growth rate and season of birth can significantly affect behaviour^25^ it is therefore possible that the between-litter variability observed in the current study, may have been partly affected by these factors.

It is well documented that although personality traits, including fearfulness and aggressiveness, are heritable^5, 6, 26^ (i.e. can be transmitted by genetic selection of specific features), early life socialisation, parental care and past experiences all play an important role in shaping the dogs’ reaction to a novel environment^25, 27, 28^. By testing pups at 8-weeks, before moving into their owner’s new homes, we aimed to reduce the effect of the environment as much as possible. However, given that a number of studies have shown effects of early handling and quality of maternal care on pups’ subsequent behaviour in testing situations, we cannot exclude the influence of these factors altogether^23, 27^.

Future research should include if possible, more stringent control of environmental effects and genetic testing to further disentangle the weight of these factors in affecting dog behaviour. Laboratories where all puppies can be reared in identical conditions offer an ideal experimental set up to control for such aspects, however, they are also limited since they can not reflect the variety of environments offered by breeders and assessing large sample sizes would involve significant ethical concerns.

It is worth mentioning that this open field test was not designed to assess the full range of behavioural expression of a dog. Some traits, such as trainability, cannot be detected by this 5-minutes open-field test, but they would need additional assessment protocols. Previous studies, for example, have reported breed differences when assessing aggressiveness in adult dogs^9, 13^; the current test did not directly measure aggressive reactions, and although a puppy could have shown aggressive behaviours toward the unfamiliar person or a stimulus, this was never the case. A more targeted test would be necessary to assess this trait, although its prevalence in 2-months-old puppies is expected to be very low or negligible^29^. Indeed, in another study, the first author^29^ recorded puppies’ aggressive reactions during a food subtraction test in only four out of 162 puppies.

Overall, our results suggest that breed selection has affected the expression of personality traits and this can be observed already at 2 months. However, results also highlight that within each modern breed there is a very high variability, even when selecting breeders from the same geographical area (i.e. northern/central Italy). This likely explains why our breed profiles did not always reflect the personality descriptions associated with that breed. For example, some personality traits appeared to be consistent with breed-club descriptions and general expectations (e.g. Siberian huskies being less exuberant and Rottweilers less cautious than average), others were rather unexpected (e.g. low playfulness in Border collies). It should be mentioned that this could also be an effect of variances in developmental trajectories of different breeds^30^ which may have had an impact on the expression of specific behaviours. Further research should take this aspect into account. In addition, while the 5-minutes open-field test procedure was standardized, we cannot exclude the possible contribution of transient differences in puppies’ motivational and activation states at the time of testing.

Indeed, the large within breed variability found among a generic sample of breeders (which more closely resembles the choice of an average buyer), highlights the importance of shifting the attention of future dog owners, from just ‘breed selection’ to a more careful assessment of the pups’ characteristics. Relinquishment of dogs to shelters may be linked to failed expectations, which could be fuelled by an inadequate/misleading view of a specific breed. Hence, increasing public awareness of the importance of visiting the breeder, asking about breeding strategies, seeing the parents of the litter and assessing individual behavioural differences among littermates are key steps to engender informed buyers.

## Conclusions

Modern breeds undergo diverse selective pressures for which the resulting behavioural characteristics might not reflect the conventional/historical and genetic categorizations of breeds^7, 8, 12^. Given the high within-breed variability recorded in this study, researchers should take care when comparing breeds, not to treat them as homogeneous groups. Furthermore, breeders and prospective owners should avoid relying solely on the general knowledge of a breed’s characteristics but rather consider directly assessing individual animals. It is worth mentioning that puppy assessments do not ensure the stability of the personality traits in adulthood^18, 31^, nevertheless, they may give some indication of the present attitude of a pup thereby helping in deciding the most suitable home for it. Further work is needed to determine if the behavioural differences found remain consistent when dogs are retested at a later stage when the individual is in a new environment.

## Methods

### Ethics statement

All procedures were performed in full accordance with Italian legal regulations and the guidelines for the treatments of animals in behavioural research and teaching of the Association for the Study of Animal Behavior (ASAB). In Italy, observational studies of animal behaviour are considered procedures not subjected to the National Directive n. 26/14 (transposition of the 2010/63/UE directive on the protection of animals used for scientific purposes, article 1, comma 5), and for those, further ethical approval is not requested. Hence, no special permission was needed to carry out this study. Nevertheless, when first visiting the breeders, an in depth description of the test was presented by the researcher and consent to video-record and use data in an anonymous form was sought verbally prior to testing.

### Subjects

We tested 377 puppies from 12 different breeds. To avoid assessing a specific bloodline, litters came from different commercial and hobby breeders (n = 51), all situated in northern and central Italy. All puppies were tested at 2 months (range 58–62 days) at the breeders’ premises before adoption. The sample was balanced for sex, and a mean of 6.2 litters per breed was tested (Table 2).

**Table 2:** Description of sample size.

Breed	Total puppies	F	M	Litters	Breeders
Alaskan malamute	23	13	10	4	3
American staffordshire	32	18	14	7	6
Argentinian dogo	30	15	15	5	3
Australian shepherd	37	20	17	6	4
Border collie	26	14	12	4	4
Boxer	40	20	20	11	6
Doberman	25	11	14	5	4
German shepherd	36	14	22	7	5
Golden retriever	33	20	13	8	6
Labrador retriever	39	19	20	8	4
Rottweiler	26	10	16	5	3
Siberian husky	30	13	17	4	3
Total	377	187	190	74	51

Total number of puppies per breed, sex, number of litters per breed and number of different breeders from which the litters came from.

### Open field test

The open field test was set up in a quiet area at the breeder’s premises. Testing was normally carried out in the morning (9–11 h), but could vary according to breeder availability.

**Figure 3:**
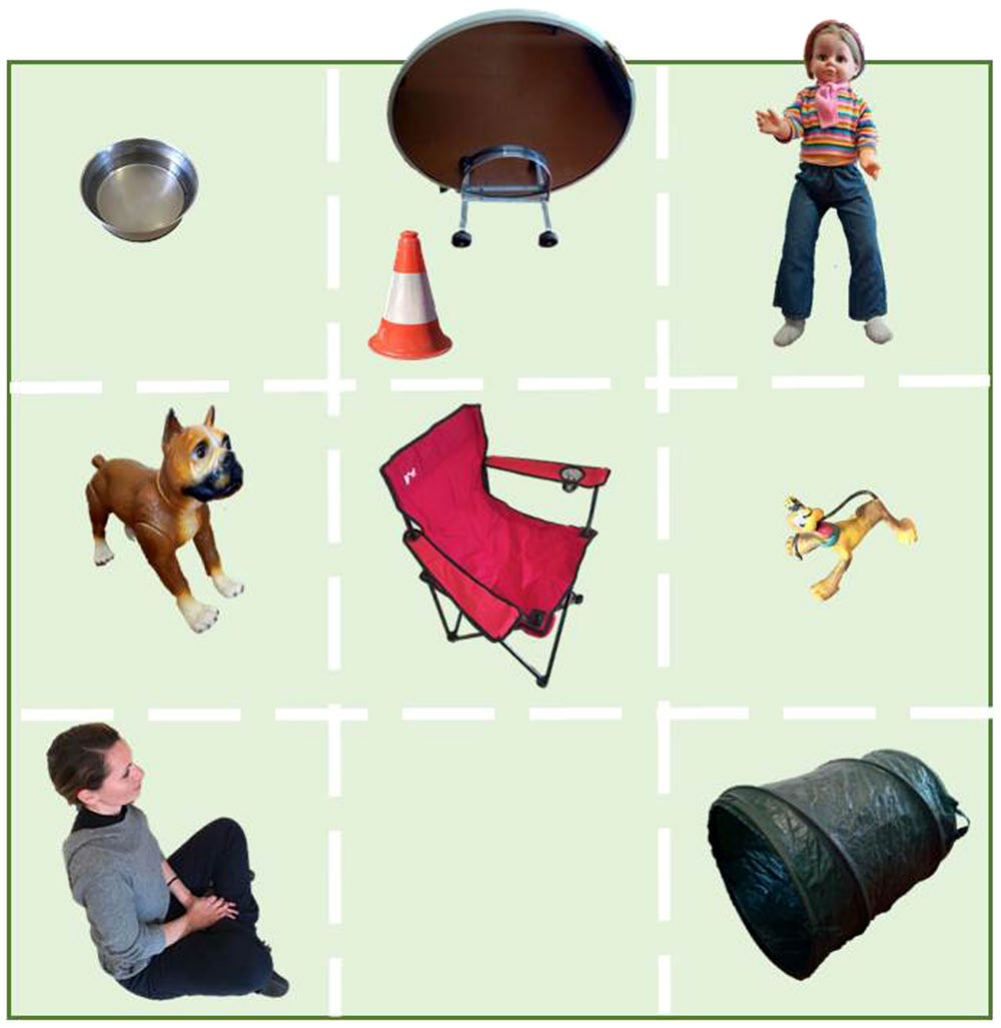
Stimuli and setup of the open-field test. From top left (1) a bowl with water; (2) a street cone and a mirror placed at puppy height; (3) a child-looking doll standing up (approx. 86 cm high); (4) a realistic looking plastic dog (approx. 50 cm tall, boxer type), displaying an erect posture; (5) the breeder seated on a chair; (6) a squeaky dog toy; (7) a female researcher (unfamiliar to the dog) seated on the ground; (8) this square was left empty; (9) a small nylon tunnel (53 cm long and 43 cm diameter) with a small piece of food placed inside. Objects are not to scale.

A 5 × 5 m arena was temporarily fenced off using a portable ‘puppy pen’ (1 m high) covered by a dimming green net (to avoid distraction from the outside). Using powdered chalk, the area inside the pen was divided into 9 identical squares each one containing a different stimulus (Fig. 3). The position of the stimuli was the same for all pups tested. The breeder and the experimenter (both sitting inside the arena) adopted a relaxed posture and remained quiet and passive during the whole test. The breeder was asked to carry the pup into the pen, and once seated, place the pup on the ground in front of his/her feet. The pup was then free to move around in the pen for 5 minutes. A video camera was set up on a tripod outside the pen, and manoeuvred by an assistant so as to insure the pup’s behaviour was recorded during the whole test.

### Behavioural coding

In total 11 mutually-exclusive behavioural categories were recorded continuously in terms of frequency and duration of their occurrence (Table 3). The ethogram used was the same developed during a previous methodological study run by our research team which proved fit-for-purpose in assessing puppies’ personality traits during an open field test^15^. The stimulus toward which the behaviour was directed was also recorded. Video analyses were carried out using behavioural event recording software (Observer XT 8.0, Noldus Information Technology, The Netherlands).

**Table 3:** Behaviours recorded during the study.

Behavioural variable	Short Description
Walk	Walking around the arena without looking/interacting with any stimulus in particular
Fast gait	Trotting, or galloping/bounsing around the arena without looking/interacting with any stimulus in particular
Cautious approach/interaction (object or people)	Hesitant approach to a stimulus, olfactory inspection with lowered posture and slow movements
Positive approach/interaction (object or people)	Direct, relaxed approach to the stimulus, sniffing or pawing it with tail hanging, held parallel or slightly above the bodyline
Exuberant approach/interaction (object or people)	Direct approach at fast gait, often dashing towards the objects and knocking them over or sniffing/licking the stimulus wagging rapidly and hurtling.
Social interaction (people)	Includes greeting behaviour (wagging rapidly, often licking the persons’ face/hands), climbing into the experimenter’s lap, lying down next to the person belly-up, and attention-seeking behaviours.
Playful interaction (object or people)	Includes play bow and other play-related behaviours (e.g. non-aggressive grabbing, pulling and biting toy, mouse jumping, predatory behaviours, carrying the toy around in the mouth)
Deflection (object or people)	The pup increases the distance from the stimulus, shows avoidance behaviours and startle response
Look at stimulus (object or people)	Visual exploration of the stimulus, the dog is oriented and looking towards it from at least a few paces away. This behaviour often occurs just before an interaction or avoidance of the object
Non-stimuli-related behaviour	This category captures the time pups spent not interacting/engaging with the stimuli (and not walking/trotting). The pup is either in a static position (sitting, lying or standing), or exploring/interacting with the environment. Also includes maintenance behaviours (i.e. drink, eat biscuit - which was in the tunnel, elimination).

Behavioural variables were measured as frequencies (f) or durations (d) of occurrences.

### Analysis

A preliminary Exploratory Factor Analysis was carried out to identify main factors of associated behaviours but the KMO (Kaiser-Meyer-Olkin measure of sampling adequacy) was too low (0.471). Thus, following the methods in Barnard, Marshall-Pescini *et al*.^15^, we performed a Hierarchical Cluster Analysis (method: average—linkage between groups; similarity measure: Euclidean squared distance), using the variables in Table 3. Deflection was discarded because it was shown by less than 30% of the subjects. To improve the homoscedasticity of variables, data were standardised using z-scores. The Hierarchical Cluster Analysis creates subsets (or clusters) of objects (i.e., observations, individuals, items of variables) such that those within each cluster have a higher degree of similarity than objects assigned to different clusters. Similarities (or dissimilarities) are defined by an appropriate metric (a measure of distance between pairs of observations), and a linkage criterion.

With the aim to investigate breed, litter and sex effects on each of the personality traits identified by the cluster analysis, we applied a Generalized Linear Mixed Model (GLMM) running a dredge model selection procedure, using the R package MuMin to identify the best model (Akaike Information Criterion). Starting from a null model, we added the random effects nested into the litter factor and the fixed effect of breed, sex and litter and their interaction. Details of the procedure applied can be found in the Supplementary Information.

To allow a general profiling and overview of our different breeds, mean and 95% confidence interval (CI) were calculated. This allowed assessing how each breed differed from the population mean on each personality trait. One-sample t-test was used to calculate significant p-values (α ≤ 0.05).

R (3.3.2) statistical programmes were used for all the analysis.

## Electronic supplementary material

Supplementary Information
