# Supplementary material for: Breed, sex, and litter effects in 2-month old puppies’ behaviour in a standardised open-field test
Source: Sci Rep. 2017 May 11;7:1802. doi: 10.1038/s41598-017-01992-x (PMC5431970; doi:10.1038/s41598-017-01992-x)
Supplement: Supplementary file 1 — Supplementary Information [file 41598_2017_1992_MOESM1_ESM.pdf]

SUPPLEMENTARY INFORMATION – ANALYSIS AND RESULTS

Breed, sex, and litter effects in 2-month old puppies' behaviour in a standardised open-field test

Shanis Barnard, Sarah Marshall-Pescini, Annalisa Pelosi, Chiara Passalacqua, Emanuela Prato-Previde, Paola Valsecchi

1. Hierarchical Cluster Analysis

The table below shows the results from the Hierarchical Cluster Analysis that allowed us to extract the six personality clusters described and represented by the dendrogram in the main paper.

The analysis extracted three main clusters (described in the main paper and highlighted in grey in Table S1) whilst the remaining three variables (i.e. playful interaction, non-stimuli related behaviour and social interaction) group only at a later stage with previously formed clusters. To choose only the relevant cluster groups, we calculated the partial agglomeration increment, which highlights when the relative distance between clusters is too high to justify an agglomeration (i.e. variables are not significantly associated). The partial increment is calculated as follows: [(k / k-1) - 1] where k is the agglomeration coefficient of cluster k (i.e. Euclidean squared distance), k-1 is the coefficient of the cluster preceding k.

Table S1 Hierarchical Cluster Analysis output showing the agglomeration of each variable at subsequent stadium, the agglomeration coefficient and partial increment.

Agglom. Stadium	Cluster agglomeration		Agglom. Coefficient	Partial Increment
	Variable/Cluster 1	Variable/Cluster 2		
1	**1** exuberant interaction	**2** fast gait	505.093	
1	**5** walk	**6** positive interaction	516.513	+ 0.02
1	**3** look at stimuli	**4** cautious interaction	565.524	+ 0.09
2	**1** exub int. + fast gait	**8** playful interaction	648.129	+ 0.15*
3	**3** look at stim. + cautious int.	**9** non stimuli related behav.	660.193	+ 0.02
4	**1** exub int.+ fast gait + playful int.	**7** social interaction	715.001	+ 0.08

5	**1** exub. Int. + fast gait + playful int. + social int.	**5** walk + positive interaction	793.648	+ 0.11
6	**1** exub. Int. + fast gait + playful int. + social int. + walk + positive	**3** look at stim. + cautious int. + non stim. related behav.	869.717	+ 0.10

* threshold for cluster selection

2. Generalized Linear Mixed Model (GLMM) model selection procedure

Statistical analysis was carried out in R (version 3.2.2 for Windows, http://www.r-project.org; R packages: lmerTest v. 2.0-30, MuMIn v. 1.15.6).

Table S2 Model selection from GLMM computed for the six personality clusters. Bold type indicates the models with the strongest support.

Response variable	Random factor	model	loglik	AICc	delta
**Exuberant attitude**	1|litter	**Litter**	-1630.5	3455.6	0.0
		**Breed**	-1833.8	3813.4	357.8
		Breed + sex	-1885.6	3827.2	13.8
		Breed * sex	-1934.7	3900.4	73.2
		Sex	-1975.6	3959.2	58.8
		Null	-1980.8	3967.6	8.4
**Cautious attitude**	1|litter	**Litter**	-1866.1	3923.5	0.0
		Breed * sex	-2196.1	4348.2	424.7
		Breed + sex	-2201.5	4434.3	510.8
		**Breed**	-2204.8	4438.7	515.2
		Sex	-2253.8	4515.8	592.3
		Null	-2259.8	4525.6	602.1
**Relaxed attitude**	1|litter	**Litter**	-1871.2	3933.9	0.0
		Breed * sex	-2155.4	4366.8	432.9
		Breed + sex	-2212.5	4456.4	522.6
		**Breed**	-2216.5	4462.2	528.4
		Sex	-2265.6	4539.2	605.4
		Null	-2272.6	4551.3	617.4
**Social interaction**	1|litter	**Litter**	-1683.5	3558.4	0.0
		**Breed**	-1946.8	3949.7	391.3
		Breed * sex	-1997.1	4025.5	467.1
		Breed+ sex	-2048.5	4105.1	546.8
		Null	-2054.5	4115.1	556.8
		Sex	-2216.5	4462.2	903.9
**Playful interaction**	1|litter	**Litter**	-1770.7	3732.8	0.0
		**Breed**	-2054.5	4165.0	432.2
		Breed * sex	-2108.1	4247.6	514.8
		Breed+ sex	-2159.8	4327.7	594.9
		Null	-2165.7	4337.5	604.7
		Sex	-2216.5	4462.2	729.5

**Non stimuli related behavior**	1|litter	**Litter**	-1875.5	3942.4	0.0
		Breed * sex	-2159.6	4375.3	432.9
		Breed + sex	-2214.4	4460.1	517.8
		**Breed**	-2216.5	4462.2	519.9
		Sex	-2270.0	4548.1	605.7
		Null	-2277.3	4560.7	618.3

For all measures, all attempts to create additive models considering both the nested effect of breed and litter, as well as the models involving sex * litter and sex+ litter effects, were fallacious because of **rank deficiency** in the fixed effects matrix. Rank deficiency in this context says there is insufficient information contained in data to estimate the model. Even if it stems from many origins, data are likely insufficient (it is impossible to estimate n parameters with less than n data points). In these circumstances, an output is still produced, but its information is highly unreliable; thus, considered only the principal effects (the significant ones are reported in Table 1 in the main paper) for discussion.

Table S3 Values of the GLMM for the fixed effects in discarded models (significant fixed effects in selected models are shown in Table 1 in the main paper)

Response variable	model		F	p-value
**Exuberant attitude**	Breed + sex	**Breed**	**1.039**	**0.411**
		Sex	1.249	0.264
	Breed * sex	**Breed**	**2.916**	**0.001**
		Sex	0.146	0.702
		Breed*sex	0.797	0.643
	Sex	Sex	1.141	0.286
**Cautious attitude**	Breed + sex	**Breed**	***1.688***	***0.064***
		Sex	0.051	0.821
	Breed * sex	**Breed**	***1.677***	***0.066***
		Sex	0.051	0.822
		Breed*sex	0.797	0.643
	Sex	Sex	0.088	0.760
**Relaxed attitude**	Breed + sex	**Breed**	**2.167**	**0.015**
		Sex	1.184	0.277
	Breed * sex	**Breed**	**3.075**	**0.000**
		Sex	0.605	0.437
		Breed*sex	1.438	0.154
	Sex	Sex	1.927	0.166
**Playful attitude**	Breed + sex	**Breed**	**2.891**	**0.001**
		Sex	0.536	0.464
	Breed * sex	**Breed**	**2.870**	**0.001**
		Sex	1.640	0.201
		Breed*sex	0.773	0.667
	Sex	Sex	0.682	0.410
**Social interaction**	Breed + sex	**Breed**	**3.368**	**0.000**

		Sex	1.464	0.227
	Breed * sex	**Breed**	**2.862**	**0.001**
		Sex	1.731	0.189
		Breed*sex	1.160	0.313
	Sex	Sex	0.719	0.397
**Non stimuli related behaviors**	Breed + sex	**Breed**	**2.891**	**0.001**
		Sex	0.536	0.464
	Breed * sex	**Breed**	**1.891**	**0.039**
		Sex	0.216	0.642
		Breed*sex	0.873	0.566
	Sex	Sex	2.335	0.127

3. Between breed differences: breed compared to the population mean

Table S4 Mean, Standard Error of the Mean (SEM) and 95% confidence interval (CI) are reported for each breed on each of the six personality traits. One-sample t-test was used to calculate if the population mean differed significantly from the overall population (α ≤0.05). Significant values are marked with an asterisk.

**CL1_Exuberant attitude (population mean ±SEM: 13.41±1.32)**						
**Breed**	**Mean**	**SEM**	**t**	**p-value**	**CI Lower**	**CI Upper**
Amstaff	12.18	3.89	-0.32	0.75	-9.17	6.71
Dogo	11.20	3.96	-0.56	0.58	-10.30	5.88
Husky	3.12	1.01	-10.18	<0.0001*	-12.36	-8.22
Alaskan	2.52	0.93	-11.70	<0.0001*	-12.82	-8.96
Aussie	15.54	3.13	0.68	0.50	-4.22	8.47
Border	18.51	5.35	0.95	0.35	-5.91	16.11
Boxer	26.65	7.60	1.74	0.09	-2.14	28.61
Rottweiler	6.01	2.21	-3.35	0.003	-11.96	-2.85
Doberman	14.68	4.34	0.29	0.77	-7.69	10.23
GSD	8.70	1.83	-2.42	0.02	-8.17	-0.72
Golden	9.38	3.72	-1.08	0.29	-11.62	3.56
Labrador	23.01	5.34	1.80	0.08	-1.21	20.41
**CL2_Cautious attitude (population mean 21.29±1.18)**						
**Breed**	**Mean**	**SEM**	**t**	**p-value**	**CI Lower**	**CI Upper**
Amstaff	36.22	4.37	3.42	0.002*	6.02	23.84
Dogo	16.27	3.03	-1.66	0.11	-11.22	1.18
Husky	24.75	4.11	0.84	0.41	-4.95	11.88
Alaskan	44.21	8.25	2.78	0.01*	5.81	40.03

Aussie	18.38	3.08	-0.94	0.35	-9.17	3.34
Border	23.33	5.13	0.40	0.69	-8.52	12.61
Boxer	15.16	2.36	-2.59	0.01*	-10.91	-1.35
Rottweiler	11.76	1.94	-4.90	<0.0001*	-13.53	-5.52
Doberman	20.56	3.20	-0.23	0.82	-7.32	5.86
GSD	17.84	3.56	-0.97	0.34	-10.68	3.78
Golden	19.20	3.49	-0.60	0.56	-9.20	5.03
Labrador	16.06	3.54	-1.48	0.05*	-12.41	1.95
**CL3_Relaxed attitude (population mean±SEM: 90.30±2.84)**						
**Breed**	**Mean**	**SEM**	**t**	**p-value**	**CI Lower**	**CI Upper**
Amstaff	77.81	6.63	-1.89	0.69	-26.02	1.04
Dogo	122.49	10.31	3.12	0.004*	11.11	53.26
Husky	69.61	11.37	-1.82	0.08	-43.98	2.60
Alaskan	100.49	10.23	0.99	0.33	-11.02	31.41
Aussie	107.72	8.52	2.05	0.05*	0.14	34.70
Border	99.05	9.76	0.90	0.38	-11.35	28.85
Boxer	100.05	10.76	0.91	0.37	-12.02	31.53
Rottweiler	80.21	9.51	-1.06	0.30	-29.68	9.51
Doberman	85.51	10.41	-0.46	0.65	-26.27	16.69
GSD	70.64	6.81	-2.89	0.007*	-33.49	-5.83
Golden	96.51	10.52	0.60	0.56	-15.24	27.66
Labrador	75.32	8.50	-1.76	0.09	-32.21	2.34
**CL4_Social interaction (population mean±SEM: 10.50±1.14)**						
**Breed**	**Mean**	**SEM**	**t**	**p-value**	**CI Lower**	**CI Upper**
Amstaff	18.64	4.33	1.88	0.07	-0.70	16.98
Dogo	9.69	2.47	-0.33	0.75	-5.87	4.25
Husky	1.92	1.20	-7.13	<0.0001*	-11.05	-6.12
Alaskan	4.27	1.83	-3.40	0.003*	-10.03	-2.43
Aussie	9.79	2.88	-0.25	0.81	-6.54	5.12
Border	7.46	2.66	-1.14	0.26	-8.52	2.44
Boxer	16.17	6.97	0.81	0.42	-8.42	19.76
Rottweiler	23.55	6.54	1.99	0.06	-0.42	26.52
Doberman	17.19	2.99	2.24	0.04*	0.53	12.86
GSD	6.39	2.60	-1.58	0.12	-9.40	1.18
Golden	4.47	1.74	-3.46	0.002*	-9.58	-2.48

Labrador	7.03	1.86	-1.87	0.07	-7.24	0.03
**CL5_Playful interaction (population mean ±SEM: 31.41±2.39)**						
**Breed**	**Mean**	**SEM**	**t**	**p-value**	**CI Lower**	**CI Upper**
Amstaff	18.79	5.32	-2.37	0.02*	-23.47	-1.76
Dogo	22.65	7.37	-1.19	0.24	-23.84	6.32
Husky	21.68	7.43	-1.31	0.20	-24.96	5.50
Alaskan	24.77	12.43	-0.53	0.59	-32.41	19.13
Aussie	33.05	6.12	0.27	0.79	-10.77	14.04
Border	12.62	3.80	-4.95	<0.0001*	-26.62	-10.97
Boxer	48.02	8.51	1.95	0.06	-0.61	33.83
Rottweiler	14.44	4.46	-3.81	0.001	-26.16	-7.79
Doberman	57.02	10.15	2.52	0.02*	4.67	46.55
GSD	44.44	10.48	1.24	0.22	-8.24	34.30
Golden	28.17	8.38	-0.39	0.70	-20.34	13.86
Labrador	39.37	7.50	1.06	0.30	-7.23	23.15
**CL6_Non stimuli related behaviour (population mean ±SEM: 102.12±3.52)**						
**Breed**	**Mean**	**SEM**	**t**	**p-value**	**CI Lower**	**CI Upper**
Amstaff	123.58	11.84	1.81	0.80	-2.69	45.60
Dogo	96.20	11.19	-0.53	0.60	-28.81	16.96
Husky	133.51	16.33	1.92	0.07	-2.06	64.83
Alaskan	114.38	11.85	1.04	0.31	-12.31	36.83
Aussie	89.38	9.80	-1.30	0.20	-32.61	7.13
Border	123.22	14.57	1.45	0.16	-8.91	51.11
Boxer	87.57	13.14	-1.11	0.28	-41.13	12.03
Rottweiler	99.58	11.39	-0.22	0.83	-26.00	20.93
Doberman	77.59	8.65	-2.84	0.009*	-42.39	-6.68
GSD	103.39	11.29	0.11	0.91	-21.65	24.19
Golden	97.37	10.38	-0.46	0.65	-25.91	16.41
Labrador	91.32	10.76	-1.00	0.32	-32.64	11.03

Breeds: Amstaff=American Saffordshire Terrier; Dogo=Argentinian Dogo; Husky=Siberian Husky; Alaskan=Alaskan Malamute; Aussie=Australian Shepherd; Border=Border Collie; GSD=German Shepherd Dog; Golden=Golden Retriever; Labrador=Labrador Retriever
